# The impact of artificial intelligence on drug discovery for neuropsychiatric disorders

**DOI:** 10.17179/excli2025-8378

**Published:** 2025-07-03

**Authors:** Vickram Agaram Sundaram, Bharath Saravanan, Bhavani Sowndharya Balamurugan, Mathan Muthu Chinnakannu Marimuthu, Kavita Munjal, Hitesh Chopra

**Affiliations:** 1 Department of Biotechnology, Saveetha School of Engineering, SIMATS, Chennai, 602105, India; 2 Amity Institute of Pharmacy, Amity University, Noida, Uttar Pradesh, India; 3 Centre for Research Impact & Outcome, Chitkara College of Pharmacy, Chitkara University, Rajpura, 140401, Punjab, India

**Keywords:** Artificial Intelligence, machine learning, neuropsychiatric disorders, blood-brain barrier, AI-driven drug development

## Abstract

Artificial intelligence (AI) and machine learning (ML) are transforming medication discovery, particularly in neuropsychiatric illnesses, where traditional drug research presents major obstacles. This paper looks at how artificial intelligence might help advance neuropsychiatric medication development, with an emphasis on early-stage research, drug design, and clinical diagnostics. This review discusses AI's contribution to understanding the blood-brain barrier and its link with the central nervous system, which is an important aspect of medication efficacy in neuropsychiatric treatments. AI-facilitated de novo drug design, using predictive algorithms and deep learning models, speeds up the discovery of new medicinal molecules. AI is employed in brain imaging and diagnosis, boosting the accuracy with which neuropsychiatric diseases are identified. BBB permeability prediction is one of the most important uses of AI in drug discovery, as it improves the selection of CNS-active drugs. Additionally, AI is transforming treatment techniques for neurodevelopmental disorders and assisting in the discovery of novel antidepressant medications through data-driven methodologies. Despite these accomplishments, AI-driven drug discovery still has several constraints, such as data biases, regulatory barriers, and ethical issues. Overcoming these restrictions will be critical to unlocking AI's full potential in neuropsychiatric research. This paper concludes with several future possibilities and opportunities, such as incorporating AI into personalized medicine using sophisticated neural network models and multimodal data fusion techniques. This might increase treatment choices for certain conditions by fine-tuning AI approaches. This paper presents a perspective on AI as a highly transformative instrument for influencing neuropsychiatric drug development, as well as an emerging field that has the potential to impact the modern idea of pharmacology.

See also the graphical abstract(Fig. 1).

## Introduction

Epigenetics, described as the study of alterations in gene functions that don't alter the primary DNA sequence but dramatically influence gene expression, comprises numerous essential mechanisms. One of such is DNA methylation, which includes the attachment of a methyl grouping to the DNA's cytosine based and often causes to the silence of the gene. Another method is histone alteration, which refers to modifications in the histones, the the proteins close to which DNA is coiled. Such alterations influence the access of genes for gene transcription and are crucial to gene expression regulating it. In addition, the regulation of short noncoding RNAs plays a key function in modulating gene expression following the transcription process. These dynamic processes are critical in diverse biologically contexts, including creation, differentiation, tissue accuracy, and reactions to environmental changes. Most significantly, they can be affected by aspects such as ecological exposure, age, and disease states. Drug research and discovery is a protracted, multidisciplinary process with little guarantee that a medicine will be successful. A foundation for the growth of scientific fields has been established by the information technology revolution. Artificial intelligence (AI) and machine learning (ML) have emerged as a result of research into contemporary drug discovery techniques (Vatansever et al., 2021[68]). These technologies can mine complexities collected by biological processes at an unprecedented level using the multitude of omics along with smart technologies in massive databases in this big data era. Due to the creation of massive data from rapid screening and computational inspection of databases for lead and target discovery, the reliability of AI/ML-based models has significantly enhanced (Gautam et al., 2023[25]). The widespread availability of contemporary graphically intensive processing units (GPUs) with tensor processing units (TPUs), which can perform trillions of processes per second with parallel processing, has also significantly increased their data processing rate. Neurological (CNS) disorders are frequently thought to be the most deadly, common, and debilitating type of sickness that affects the brain in different ways. Developing new CNS medications frequently calls for specialists to be adaptable and creative, using multidisciplinary approaches from the very beginning of the drug development process (Dhankhar et al., 2024[19]). In this big data era, innovative tools and methods that can speed up pharmaceutical research are desperately needed to deliver the finest solutions for lowering failures and consistent CNS medication development. The creation of algorithms based on artificial intelligence and machine learning, which can extract characteristics or patterns from massive, intricate datasets produced by the drug discovery process, is essential to this change (Dorahy et al., 2023[22]). 

AI/ML techniques have demonstrated state-of-the-art performance in tackling a variety of issues in discovering and creating drugs and are frequently used in several fields of data-driven decision-making. Although significant progress has already been achieved in comprehending the composition and operation of CNS illnesses, much work remains in the development of CNS medications and the clinical course of those treatments (Farghali et al., 2021[23]). There are numerous reasons why CNS medications fail preclinical and clinical trials, including the blood-brain barrier, adverse effects nausea, vertigo, and seizures, inadequate target selection, and a lack of knowledge about complicated CNS illnesses. In both preclinical and clinical studies, CNS medications have a greater likelihood of failing than non-CNS medications; for disease-modifying therapy, the failure rate has reached 100%. 

Disorders affecting the brain and the nervous system in general are referred to as neurological disorders. Alzheimer's disease (AD), Parkinson's disease (PD), multiple sclerosis (MS), brain tumors, stroke, migraine, epilepsy, and traumatic disorders are some of these conditions. More than two-thirds of dementia cases in adults 65 years of age or older are caused by Alzheimer's disease, the most terrible class of neurological illnesses (Costamagna et al., 2021[16]). Lack of the neurotransmitter dopamine causes Parkinson's disease (PD), a chronic, progressive neurological illness. Multiple sclerosis, epilepsy, stroke, migraine, and traumatic illnesses are among the numerous types of neurological disorders. Over 6 million people have strokes each year, and over 80% of these fatalities occur in low- and middle-income nations. Therapeutic techniques have not yet been used to treat the aforementioned neurological illnesses; instead, abstract shows AI speeds up the process of finding new medications for neurological conditions by evaluating clinical and genomic data, finding promising medications, and maximizing their effectiveness. Faster market rollout, safety, and regulatory compliance are all guaranteed by the procedure and have only been used in diagnostic-based approaches. Thus, scientists must focus on the different kinds of illnesses and develop novel medications or inhibitors that target the proteins implicated in this field (Patel et al., 2021[50]). 

## Artificial Intelligence's (AI) Function in Drug Development

Fundamental ethical norms, including transparency, equality, and accountability, must guide AI's deployment in clinical studies. Compliance with rules such as the General Data Protection Regulation (GDPR) and the Health Insurance Portability and Accountability Act (HIPAA) is vital for preserving patient privacy . GDPR, with its strong data protection regulations, influences worldwide AI management and sets a bar for data privacy. Data security and privacy are essential in clinical research. The FDA describes AI as a wide category containing algorithms and models able of learning, making decisions, and prediction. ML, a subtype of AI, develops models through information analysis. The FDA's discussion paper defines an involvement to a risk-based regulation approach that supports innovation while promoting patient safety. It covers a wide array of drug growth activities-from initial discovery to post-market surveillance-and evaluates how AI/ML might be applied across various stages, including drug discoveries, non-clinical and clinical investigation, post-marketing safety tracking, and developed manufacturing. While understanding the potential advantages of AI and ML, the FDA additionally addresses the concerns associated with these advancements, such as data biases and constrained explainability of models. The entire drug-discovery process might be drastically changed by utilizing machine learning and artificial intelligence (AI) algorithms, which would have numerous benefits. The most notable of them is AI's capacity to quickly and effectively screen large compound libraries, greatly enhancing the discovery of possible therapeutic candidates. Furthermore, by forecasting the safety and effectiveness profiles of potential medicines, AI systems can provide priceless insights and lessen the need for intensive preclinical and clinical research's predictive ability might expedite the drug development process and increase clinical trial success rates, which would ultimately lead to the production of safer and more effective medicinal medicines (Moingeon et al., 2022[45]). However, some issues need to be addressed when using AI in drug discovery. The necessity of obtaining diverse and high-quality data is a major obstacle. Additionally, maintaining the comprehension of AI models is crucial for gaining regulatory approval and fostering confidence in the scientific and medical communities. Another significant obstacle that calls for careful navigation is addressing ethical issues, such as data privacy and prejudice mitigation (Rai et al., 2023[53]). Table 1(Tab. 1) (References in Table 1: Bhatt et al., 2024[8]; Sun et al., 2023[64]; Terranova et al., 2024[65]) explains the neurodegenerative conditions with its current markers for diagnosis.

### Initial efforts to determine the BBB's presence and its connection to the neuronal system (CNS)

Treating diseases that affect the brain and spinal cord is made more difficult by the blood-brain barrier (BBB), which restricts the entry of drugs into the brain's central nervous system (CNS). These days, research is focused on developing novel approaches to cross the blood-brain barrier and treat conditions that impact the central nervous system (Abd-Alrazaq et al., 2022[1]). The integration of all sensations detected by peripheral nerves and the synchronization of reactions to those feelings are the functions of the nervous system's center (CNS), which is made up of the brain and spinal cord (Doherty et al., 2023[21]). In addition to these functions, the brain and spinal cord are the most vital organs in humans, and as such, shielded by several structures, including the blood-brain barrier (BBB), meninges, bones, and cerebrospinal fluid (CSF). Molecules are transported passively between cells from their more concentrated side to their less concentrated side. Because endothelial cells have tight connections, it is tightly controlled (Vicidomini et al., 2024[70]). It's quite hard to create new molecules that can use this pathway, thus when researchers want to disperse drugs between two cells, employ another material that leaks through tight junctions, Claudin-5-Binders. Passive transport transfers molecules between the region of the blood-brain barrier are more concentrated to the side where are less concentrated; however, in this instance, the transport occurs across the cells rather than between them Kashyap et al., 2021[36]). Only tiny lipophilic medications (such as steroids) that fit the following criteria can do so. Essential chemicals amino acids and glucose are transported to the brain via this pathway, but any molecule that resembles glucose or those amino acids may be advantageous. Receptor-mediated transcytosis is another name for this process, which uses vesicles created when molecules attach to a particular receptor to transport them across the blood-brain barrier. This is true for large macromolecules such as lipoproteins, insulin, and transferrin. Polycationic compounds, sera or other peptides, might employ this pathway's non-specific transcytosis to embed themselves in vesicles after contacting with endothelial cells' negative surface. Transcytosis allows cells-typically immune system cells-to pass straight through the blood-brain barrier. These cells are occasionally employed as "trojan horses" to transfer chemicals into the brain, such as in viral infections (Charveriat et al., 2021[11]).

### AI in Neuropsychiatric Drug Development

The ramifications of these epigenetic pathways are particularly deep in the disciplines of neurology and psychiatric In neurological conditions including Alzheimer's, Parkinson's, and Huntington's diseases, epigenetic modifications are crucial to disease initiation, and progression, and may possibly serve as targets for therapeutic intervention. Similarly, in psychiatric diseases such as depression, schizophrenia, and bipolar disorder, epigenetic modifications are believed to play a substantial role in illness exposure, especially in the context of the interplay within genetic and environmental risk factors. This epigenetic understanding is not restricted to isolated disorders; there is accumulating evidence suggesting that common epigenetic paths may explain the relationship of neurological and mental conditions. Alzheimer's disease (AD) is a degenerative neurological disease that typically affects older adults. It is characterized by behavioral abnormalities, memory problems, and cognitive deterioration. As the leading cause of dementia, it is responsible for 60-80% of cases (Qiu et al., 2024[52]). One pathogenic feature of AD is the formation of tau tangles that beta-amyloid plaques in the brain and nervous system, which damage neurons and result in synaptic disorders (Kalani et al., 2024[35]). These changes to the brain lead to a reduction in mental abilities, such as speaking, memories, and administration, which eventually makes it more difficult to carry out daily tasks. Intelligent technology (AI) is revolutionizing contemporary medical care with its state-of-the-art abilities for predicting patient outcomes, personalizing treatment plans, and increasing diagnostics efficiency (Corrales-Hernández et al., 2023[15]). Technologies powered by AI use vast amounts of healthcare data, such as hereditary, neurological imaging, and healthcare information, to produce findings that are superior to humans' analytical capacity. The primary objective of machine learning (ML), a subfield of neural networks (AI), is to create computer algorithms that get better on their own with practice. These tools use data analysis to find patterns in datasets (Zhang et al., 2023[79]). In machine learning, supervised training uses labeled data to match input data with the correct output. It works especially well for tasks classification and regression. Artificial neural networks (NN), Bayes networks, support vector algorithms (SVM), decision tree models, random forest models (RF), and K-nearest-neighbors networks are common techniques in supervised learning. Deep computing (DL), an extremely specialized area of artificial intelligence and computational neuroscience, simulates how a human mind solves issues by analyzing data. A networked system is formed by the interconnection of nodes in each of its several layers (Rudroff et al., 2024[54]). Neuroimaging methods including computed tomography (CT), positron emission tomography (PET), and magnetic resonance imaging (MRI) are essential for detecting AD when paired with ML and DL because show both structural and functional changes in the brain. AI enhances existing photographic techniques by merely improving picture processing efficiency and accuracy, which enables faster and more accurate identification of AD-related changes. Machine learning algorithms are also capable of analyzing large genomic and proteomic data sets to identify biomarkers associated with AD, aiding in early detection and customized treatment regimens. Table 2(Tab. 2) (References in Table 2: Arrué et al., 2022[5]; Calderone et al., 2024[10]; Cheng et al., 2024[14]; de Thé r et al., 2023[18]; Kakoti et al., 2022[34]; Malikr et al., 2023[42]; Paul et al., 2024[51]; Verma et al., 2022[69]) explains the role of artificial intelligence in neurology. AI also improves cognitive and personality tests by providing tools that improve the precision of neuropsychological tests and analyze speech and communication patterns for early signs of dementia (Brady et al., 2023[9]).

### AI/ML applications in de novo drug design

The processes of toxicity, excretion, metabolism, distribution, and adsorption are all included in ADMET. ADMET characteristics are essential for both drug design and screening. According to estimations, 50% of all clinical failures are thought to be significantly caused by ADMET characteristics. Given the existence of numerous medications with established pharmacokinetic characteristics, ADMET prediction systems have advanced considerably (Mosquera et al., 2024[46]). Furthermore, AI/ML, structure-based modeling, data mining, and HTS are now included in the realm of ADMET forecasts. The effectiveness of machine learning (ML) in forecasting ADMET (absorption, distribution, metabolism, excretion, and toxicity) to speed up the identification of small compounds has been demonstrated in numerous research (Malandraki-Miller et al., 2021[41]). To generate high-quality models that offer enhanced accuracy and insightful ADMET reaction prediction based on chemical structure data, researchers have employed AI/ML algorithms. De novo drug design is the process of developing novel chemical compounds with certain biological and chemical properties that are both safe and efficient. Both cost-effectiveness and efficiency are maintained. Previous periods have seen a greater usage of AI/ML for de novo discovery (Kokudeva et al., 2024[39]). The use of AI/ML in molecular design has attracted a lot of interest. Recurrent neural networks (RNN), variational autoencoders (VAE), and adversarial autoencoders (AAE) have all recently employed de novo molecule-generative models with an ML framework (Nguyen et al., 2024[47]). One method that shows great promise for gleaning information from big collections of SMILES strings is the Recurrent Neural Network (RNN). Using new frameworks to produce unique molecules depicted, it can construct ligands that adhere to the same protocols as those in the training set template (Umbricht et al., 2024[67]).

### AI in Brain Imaging and Diagnostics

Drug combinations are commonly used to treat complex problems such as cancer, diabetes, cardiovascular disease, and neurological disorders. Increasing efficacy, reducing toxicity, and preventing the development of resistance are the objectives of concurrent medication delivery throughout treatment (Yadav et al., 2024[77]). Pharmacological interactions can be divided into three categories: synergistic, antagonistic, and additive. Drug synergy is when multiple medications combine to provide an impact that is greater than the total of their separate effects (Visan et al., 2024[72]). Unlike synergism, the combined medicinal activity of an antagonism is less than the combined reaction of the separate medicines. Lastly, when one treatment's effect does not overshadow or increase the efficacy of the others, a pharmacological combination is said to be addictive. Even if combination therapy has benefits over monotherapy, the creation of a new (Mathew et al., 2022[43]). Finding a pharmaceutical combination regimen that works well in clinical settings is still difficult. There is an inherent risk of patient harm under these circumstances, and it is not practical to consider every potential combination. The efficiency of conventional combinatorial therapy procedures is being improved by the application of artificial intelligence and machine learning algorithms to examine particular drug combinations and look into broader areas of combination treatment. When medications are used simultaneously, both boosted effects and unanticipated unfavorable side effects. There is a chance that certain harmful drug interactions could be lethal in very rare circumstances. To forecast the likelihood of adverse effects brought on by drug-drug interactions, AI/ML models have been created. The use of GCN, DNN (Deep Neural Networks), and ML architectures showed promising outcomes in terms of predicting adverse medication interactions (Xiong et al., 2022[76]). In contrast creating entirely new medications from scratching drug repurposed, also known as drug shifting, is the process of finding innovative applications for medications that have already been approved. 

**Figure 2:**
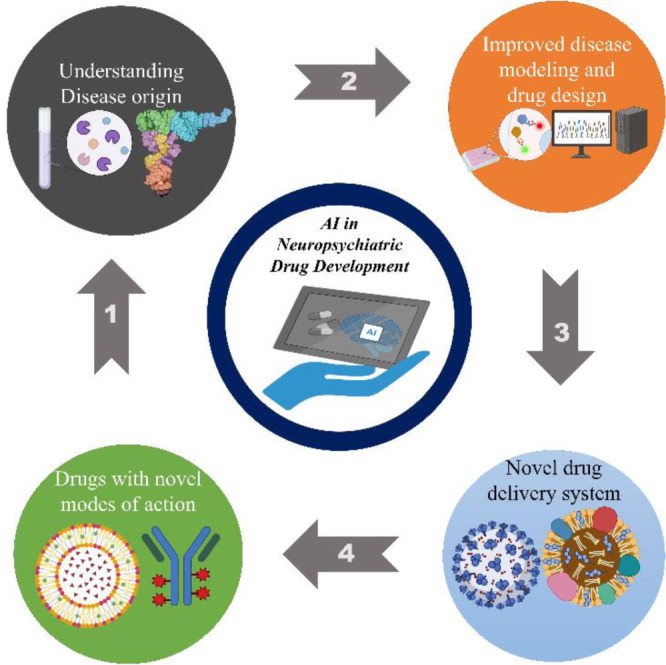
AI in neuropsychiatric drug development: a comprehensive workflow

Incorporating data from multiple sources, such as genetic, genomic, transcriptomic, chemical in nature, and bioactive data, allows computer science's sophisticated techniques to uncover possible medical applications (Gupta et al., 2023[31]). These algorithms have effectively repurposed hundreds of legal drugs, even if the underlying pharmacology and biology are complex and unknown. The following types of AI/ML applications are used for medication repositioning: (i) Use of a variety of classifiers, including CNN, SVM, RF, KNN, logistic regression, and others, in similarity-based methods. (ii) Network-based methods mostly use semi-supervised learning algorithms, whereas feature vector-based methods use both supervised and semi-supervised learning algorithms (Zhang et al., 2021[80]). By comprehending the causes of diseases, enhancing modeling of diseases and drug design, developing innovative ways to deliver drugs, and identifying medications with distinct modes of action, artificial intelligence (AI) revolutionizes the creation of neuropsychiatric medications (Fig. 2(Fig. 2)). It makes the entire process more efficient for more potent medicines.

### AI-Driven Drug Discovery for Neuropsychiatric Disorders

**Figure 3:**
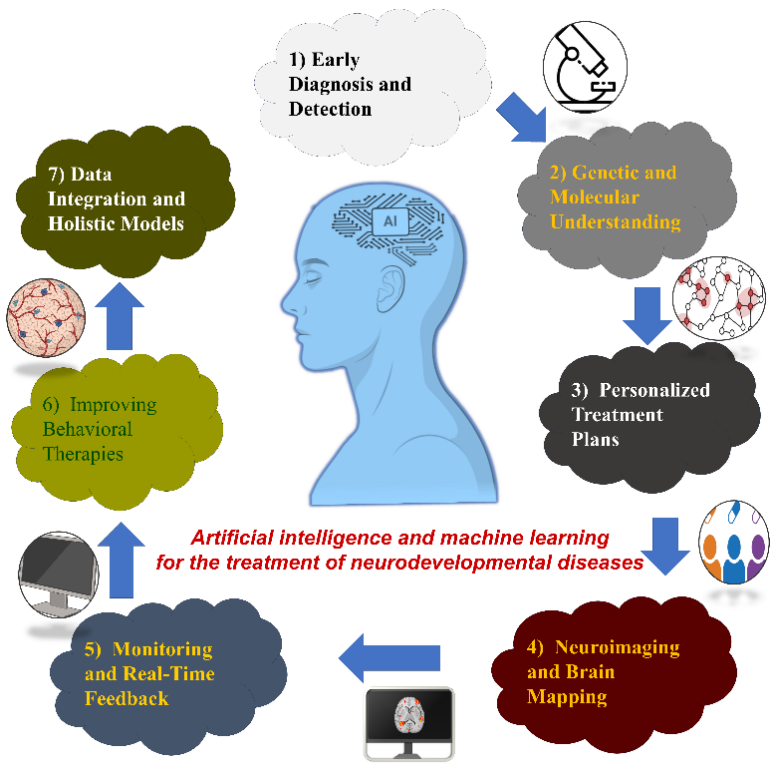
Intelligent computing helps advance the recognition and management of neurologically derived disorders

Drug studies centered on machine learning have increased because of the availability of multi-omics information and its exceptional. Components for computers, graphic processing units (GPUs). Significant success has been achieved by academic and corporate researchers in the use of artificial intelligence (AI) to create medications for AD or ADRD (AD-related psychosis). CNS diseases are a group of neurological disorders that significantly impact society and the economy. The complexity of brain structure and function, the existence of the blood-brain barrier, and our incomplete understanding of the biology behind these complicated disorders make it more challenging to create novel drugs for CNS disorders than for other diseases. This section offers an overview of AI/ML-based approaches to problems such as BBB permeability in CNS drug discovery (Gupta et al., 2022[29]). Neurological disorders and their treatment are changing as a result of computational intelligence (AI) and algorithms for learning (ML). It illustrates how AI is systematic included into all of the stages of understanding, diagnosing, and treatment (Fig. 3(Fig. 3)).

### BBB permeability prediction

One significant challenge is developing new substances that are capable of passing through the blood-brain barrier (BBB), in addition to the difficulties in determining suitable CNS targeting (Parvatikar et al., 2023[49]). The BBB serves a critical function in protecting the brain from circulating microorganisms and fluctuations in blood composition (such as hormones, potassium, amino acids, etc.). The BBB is made up of pericytes, astrocytic end-feet, microglial cells, and structural proteins (such as collagen and laminin) that form the basal lamina lining the capillary endothelial cells (Benchoua et al., 2021[7]). This biological membrane allows certain molecules to pass through via passive diffusion, facilitates the efflux of small molecules and unnecessary amino acids from the CNS back into the bloodstream, and enables the intake of water, glucose, and essential amino acids. High BBB permeability is necessary for pharmaceuticals to function well in the central nervous system (CNS), while low BBB penetration is ideal for periphery therapies to reduce negative impacts on the brain. For this reason, evaluating a drug applicant's BBB permeability early in the process of discovering drugs is essential to increasing the achievement rate of CNS medication discovery (Shukla et al., 2021[61]). 

To reduce the time, expenses, and difficulties involved in determining the permeability of the blood-brain barrier in CNS drug development, models of prediction based on machine learning (AI) have been constructed and created recently (Ganesh et al., 2022[24]). To construct these predictive models, researchers have used a variety of supervised learning techniques, concentrating on characteristics molecular size, hydrophilicity (as determined by ClogP), lipophilicity (as determined by ClogD), topological polarized surface area, the total number of basic as well as acidic atoms, donor molecules of hydrogen bond or supporters, water-accessible volume, movable bonds, and their van der Waals responses volume, along with other chemical and physical characteristics (Moingeon et al., 2023[45]). Nevertheless, all of these methods rely on a small number of biological descriptors and have predictive power mainly for passive diffusion. Some chemicals, such as insulin and glucose, can't be sufficiently explained by simple physicochemical qualities alone since the blood-brain barrier through intricate pathways involving particular drug-transporter and drug-receptor interactions. Furthermore, the CNS's therapeutic medication concentrations are restricted by membrane transporters such as the ATP-binding cassettes and the efflux transport P-glycoprotein (P-gp). These efflux transporters can limit the entry of numerous drugs and lead to CNS pharmacy resistance, even though their goal is to stop neurotoxins, which are from entering the brain (Visibelli et al., 2023[73]). Therefore, in addition to physicochemical characteristics, prediction models must take into consideration several mechanisms about how medications traverse the blood-brain barrier and continue to function in the brain. Through the integration of molecular fingerprints and physical properties, scientists developed an SVM (support vector machine) model that links passive diffusion to particular interactions such as protein interactions and uptake. The accuracy of this method was higher than that of the current SVM-based permeation of the BBB predictions, indicating that physicochemical characteristics and biochemical fingerprints can be combined to generate more accurate estimates (Gopinath et al., 2023[27]).

All AI/ML-based models that have been explored up to this point, however, have only been based on molecular features, ignoring other important information on the effectiveness of CNS drugs. Although multiple clinical trials for drug candidates generate extensive phenotypic data within the CNS, the link between the adverse effects of these medications and their BBB penetration remains insufficiently documented. A new approach has been developed to estimate BBB permeability based on the clinical characteristics of drugs, including their indications and side effects (Voigtlaender et al., 2024[74]). Although this SVM approach has brought in an alternative perspective by taking into account the diffusion of passively and potential benefits of active transport, its ability to predict still needs to be increased. Pharmaceutical side effects and medical results have an intricate connection, underscoring the different roles played by both chemistry and physics. Traditional categorization methods may not effectively explore the relationship between data and outcomes. In contrast, deep learning (DL) architectures can extract valuable insights from complex datasets with abstract associations. Consequently, a DL model was established to predict the BBB permeability of medications based on clinical characteristics, and it has outperformed existing approaches (Goles et al., 2024[26]).

## Challenges in AI-Driven Drug Discovery for Neuropsychiatric Disorders

To generate more effective therapies and stay comparable with new high-tech drugs neuroscience, gene, and stem cell therapy, neuropsychiatric drug development should focus on non-neurotransmitter-related desires actors of epigenetics and synaptic plasticity. Although a useful and often used anti-seizure drug, valproic acid (VPA) is teratogenic when used during pregnancy, therefore influencing brain and spinal cord development for reasons mostly unknown. Here, we developed a genetic recombinase-based SOX10 reporter system in human pluripotent stem cells allowing monitoring and lineage tracing of Neural Crest cells (NCCs) in a human organoid model of the embryonic neural tube. We discovered that VPA causes significant cellular senescence and drives mesenchymal differentiation of human NCCs. Cognitive and mental illnesses are treated symptoms using medications. The discovery and development of so-called disease-modifying therapies-those that can slow, stop, or even reverse the progression of the disease-is a continual effort. While a fully preventive intervention would be desirable, this would entail a remedial treatment. However, there are certain limitations to the practicality of pharmacological prophylaxis for these conditions (Chaves et al., 2021[12]). Table 3(Tab. 3) (References in Table 3: Abuasal et al., 2022[2]; Gupta et al., 2023[31]; Hayat et al., 2021[32]; Iqbal et al., 2023[33]; Nosrati et al., 2023[48]; Sahu et al., 2022[56]; Saifi et al., 2024[57]; Srivastava et al., 2021[63]; Xiong et al., 2023[75]; Yao et al., 2024[78]) describes the Chosen research employing neural organoids to simulate neurological disorders.

### Artificial intelligence and machine learning for the treatment of neurodevelopmental diseases

There is a growing consensus on AD, which concludes the disease's pathogenic events start years before symptoms appear. Regular examinations for diagnosis, beginning at a young age, should be the initial step towards a future preventative treatment (Leite et al., 2021[40]). This presents an extra obstacle to be conquered, but even though it will be resolved, the query still stands as to whether people who are at risk-not necessarily-would choose to begin a lifetime pharmaceutical therapy, which carries some risk in and of itself-to avoid getting sick twenty to forty years later. There is yet hope for better, more efficient symptomatic or therapeutic medicines because it will take numerous decades to reach the point where this problem might arise (Askin et al., 2023[6]). However, in the realm of neurological diseases, there is at least the potential for preventative medication. The issue is considerably more complex with neurodevelopmental conditions such as ASD, ADHD, and schizophrenia. The first pathomechanism in schizophrenia is a disruption in the construction of the cytoarchitecture in the embryonic stage, followed by excessive pruning in prepuberty. A brain that is unconnected and miswired is the end outcome. The current pharmaceutical treatments are post hoc in nature, and only compensating or symptomatic medications may be used because the obvious symptoms manifest months later (Kim et al., 2024[38]). Current initiatives seek to identify the disease's prodromal stage as soon as possible (around or immediately following its second hit) and develop suitable pharmaceutical and psychosocial therapies that, when administered at this time, may stop the progression to the first psychotic episode. The last scenario encounters the same challenges as ASD, in which there is only "one hit" early in the embryonic stage, leading to a miswired brain. Although the signs of autism manifest between the ages of 1.5 to 3, early diagnosis is challenging due to the inherent diversity in child development (placental pathologists, a newly developed diagnosis technique, may also be helpful) (Selvaraj et al., 2021[60]). Given that the triggering "hit"-the traumatic event-is easily identifiable, PTSD might be the mental illness most appropriate for preventive treatment. Every instance should require post-trauma psychological assistance, and as part of it, victims' stress and resilience to stress could be assessed. That would enable the "at risk" persons to be identified, and preventative measures could begin.

However, as was previously stated, psychological rather than pharmaceutical techniques should be used for this. The latter would only make sense if the person is developing genuine PTSD (Saldívar-González et al., 2022[58]). 

### Application of AI and ML in the identification of antidepressant medications

**Figure 4:**
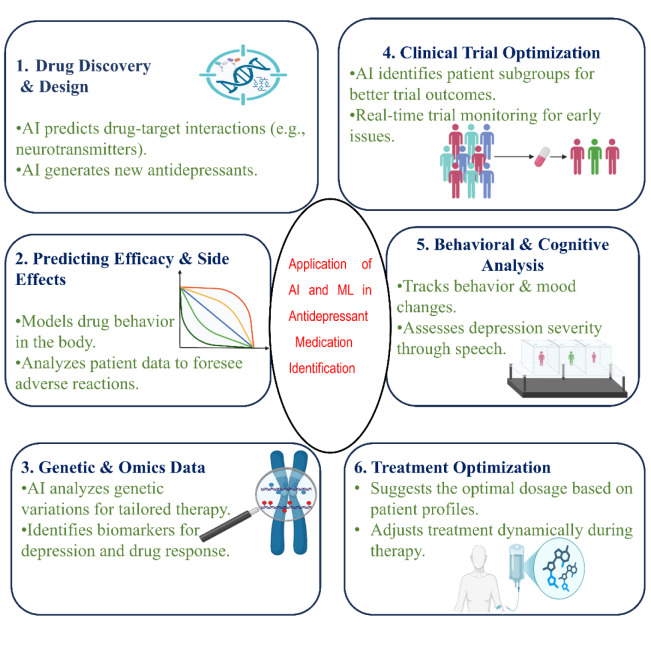
Application of AI and ML in antidepressant medication development and optimization

In de novo pharmaceutical design, novel compounds with desirable chemical and biological properties are created from the ground up to accomplish certain safety and effectiveness profiles as quickly and cheaply as possible. The automated creation of unusual chemical compounds with appropriate features has been proven possible by sophisticated AI/ML-based technologies. The use of AI/ML in de novo discovery has gained popularity in recent years due to these accomplishments. In particular, there has been a lot of interest in AI/ML-based generative molecular design. With an emphasis on generative models, AI/ML techniques are used in de novo drug design (Vilhekar et al., 2024[71]). The issue with the pipeline depicted above is that most of its mechanisms of action have previously failed multiple trials, indicating insufficient efficacy. Pharma R&D should look for strategies that could provide more potent points of therapy in degenerative and developmental illnesses that cause cognitive loss. One potential solution to this problem, particularly with the neurotransmitter-based strategy, is to boost treatment effectiveness by integrating two or three known beneficial properties into a single molecule (also known as "designed numerous ligands" or "multitarget-directed drugs") or in an assortment of drugs (Alves et al., 2022[4]. However, pharmaceutical chemistry faces a challenging task in designing and combining molecules that have a specific predetermined set of pharmacological actions while lacking several others and maintaining drug- properties, while conducting clinical studies with combinations of drugs is an acknowledgedly hard undertaking. Moreover, it is not possible to predict in advance if the pharmaceutical interaction of improved mechanisms will be advantageous (Terranova et al., 2024[65]). It becomes increasingly challenging to get the intended result with more competitors, and even preclinical studies of multi-targeted medications or combinations may quickly become too difficult and time-consuming. However, eight additional compounds are undergoing clinical research, and a fixed-dose combination of memantine and donepezil has already been introduced for intermediate-severe AD-related dementia (Al Kuwaiti et al., 2023[3]). Much work has also been done with the planned multiple ligand approach, but none of the trials have been successful and only a small number of members of this biological species have made it to the clinic. Even so, neurotransmitter-based drugs are having poor efficacy and merely provide symptom therapy, especially with the multitarget approach (Dichiara et al., 2024[20]). Drug development should be directed toward more powerful, innovative targets. Here, emphasize two of the most promising kinds: protein players in synaptic development and genomic alteration by non-coding messenger RNAs (ncRNA) (Davis et al., 2021[17]). From medication discovery and effectiveness prediction to genetic evaluation, trials optimization, behavior monitoring, and customized treatment modifications, artificial intelligence and machine learning improve the development of antidepressants (Fig. 4(Fig. 4)). These developments guarantee safer and more efficient treatments.

### Future Prospects and Innovations

Scientifically generated functional ultrasound (pharmaco-fUS) and pharmaceutical functional MR imaging (pharmacoMRI), are two new neuroimaging methods that offer in vivo functional data of particular drug effects on the brain. Despite being a proven method that is still helpful in neuropharmacology, pharmacoMRI has several difficulties (Guo et al., 2024[28]). Its preclinical usage is complicated by its limited sensitivity, anesthesia requirement, and blood oxygenation-level dependent imaging. Through the local tracking of cortical blood volume dynamics at an extraordinary spatiotemporal precision without the bias of anesthesia, pharmacy-fUS, a more recent technology, makes cerebral imaging possible. While the BBB is a static structure, current AI/ML prediction systems have largely disregarded how CNS disorders affect it (Sah et al., 2024[55]). Therefore, a model that predicts BBB penetrating using data from non-CNS disorders could not apply to CNS illnesses. Disease-related alterations in this barrier must be taken into account to increase the precision of predictions about BBB permeability. The development of disease-specific AI/ML instruments to aid CNS drug discovery is made possible by these factors. The fact that CNS drug discovery is intrinsically uncertain and involves several biological processes and pathways that cannot be boiled down to an easy sum of their functions is crucial (Sivalingam et al., 2024[62]). Drug response is influenced by several factors, including the medication's membrane permeability and the patient's genetic profile. The majority of medications show distinct effects through different biological processes. The inherent uncertainty of developing drugs for the central nervous system presents difficulties, particularly when AI/ML systems produce chemicals that might not be synthesized or forecast targets for treatment that neuroscientists know could have detrimental consequences on the brain. It is necessary to incorporate human experience and a hypothesis-driven methodology into the machine learning and artificial intelligence design procedure to overcome these issues and enhance (Khoo et al., 2024[37]). Enhance the algorithmic learning process by imparting information from human experts to AI systems. This will improve decision-making, which is an essential stage in the creation of CNS drugs. 

## Conclusion

The most recent drug development applications for CNS disease treatment are aided by AI and ML. In recent years, these kinds of uses have expanded rapidly due to the unmatched achievements of AI/ML-based techniques across several scientific as well as technological domains . The use of AI/ML will become more prevalent in CNS drug discovery in the direction of personalized healthcare in future years, particularly in these five areas; patient subtyping; identification of significant disease drivers; predicting drug response specific to cell type; independent design of new drugs; and specific to the disease cell permeability of the BBB testing. The significance of AI/ML is now being limited by structural limitations in information and algorithms. Ongoing and fresh developments in AI/ML techniques to neuroscience, however, will eventually enable us to create medications for CNS disorders that are more efficacious.

## Notes

Vickram Agaram Sundaram and Hitesh Chopra (Centre for Research Impact & Outcome, Chitkara College of Pharmacy, Chitkara University, Rajpura, 140401, Punjab, India; Email: chopraontheride@gmail.com) contributed equally as corresponding author.

## Declaration

### Declaration of competing interests

The authors declare that they have no competing interests.

### Authors' contributions

VAS - Supervision and validation; BS - Conceptualization and manuscript writing; BSB & MMCM - Conceptualization and manuscript writing; KM & HC - Methodology;

### Using Artificial Intelligence (AI)

No artificial intelligence tools were used. All content was developed and written solely by the authors.

## Figures and Tables

**Table 1:**
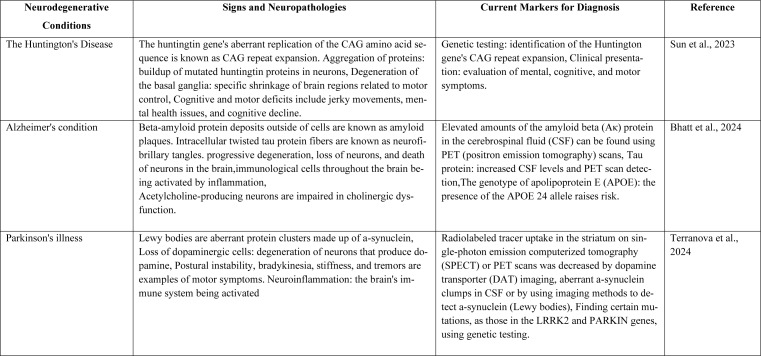
Summary of the symptoms, neuropathology, and current diagnostic indicators for the specified neurodegenerative diseases

**Table 2:**
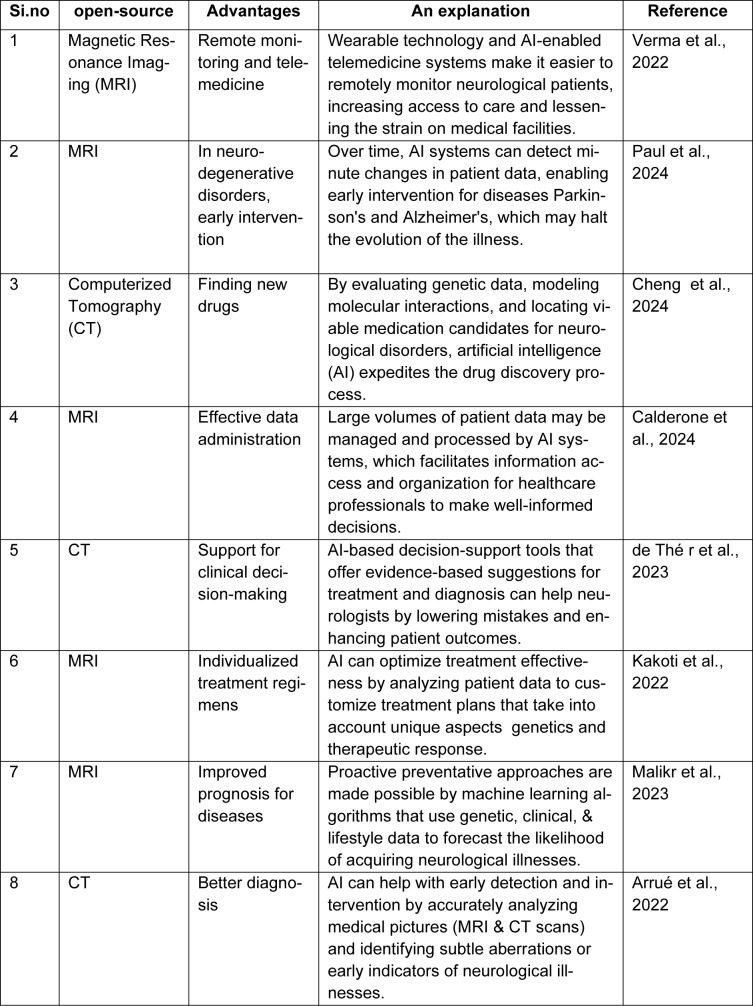
Artificial intelligence (AI) advantages for neurology

**Table 3:**
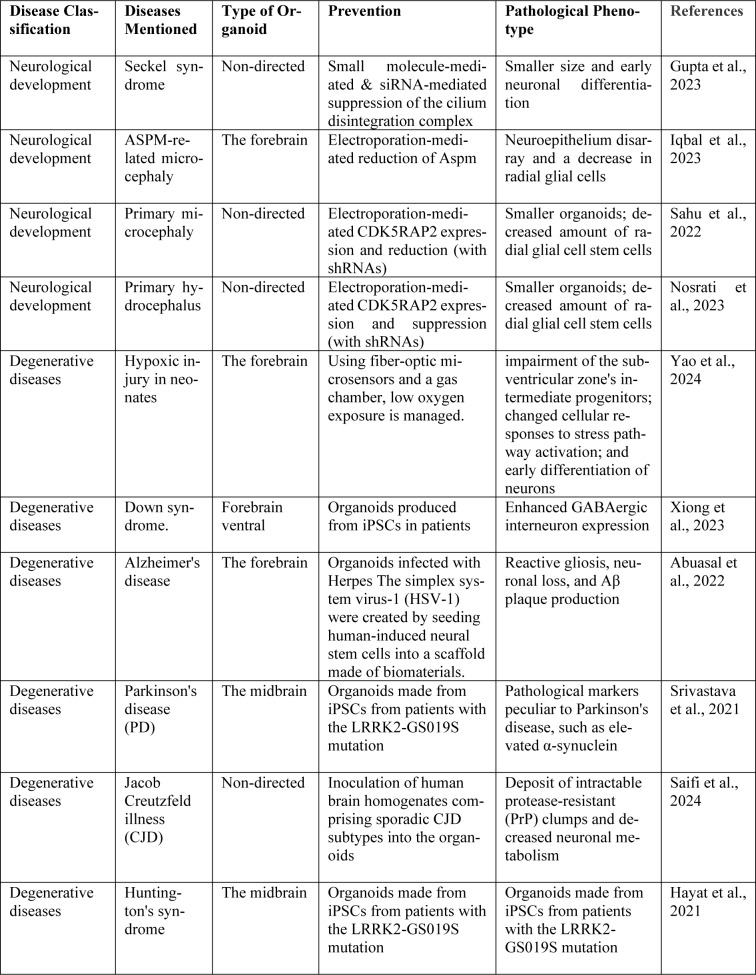
Table 3. Chosen research employing neural organoids to simulate neurological disorders.

**Figure 1:**
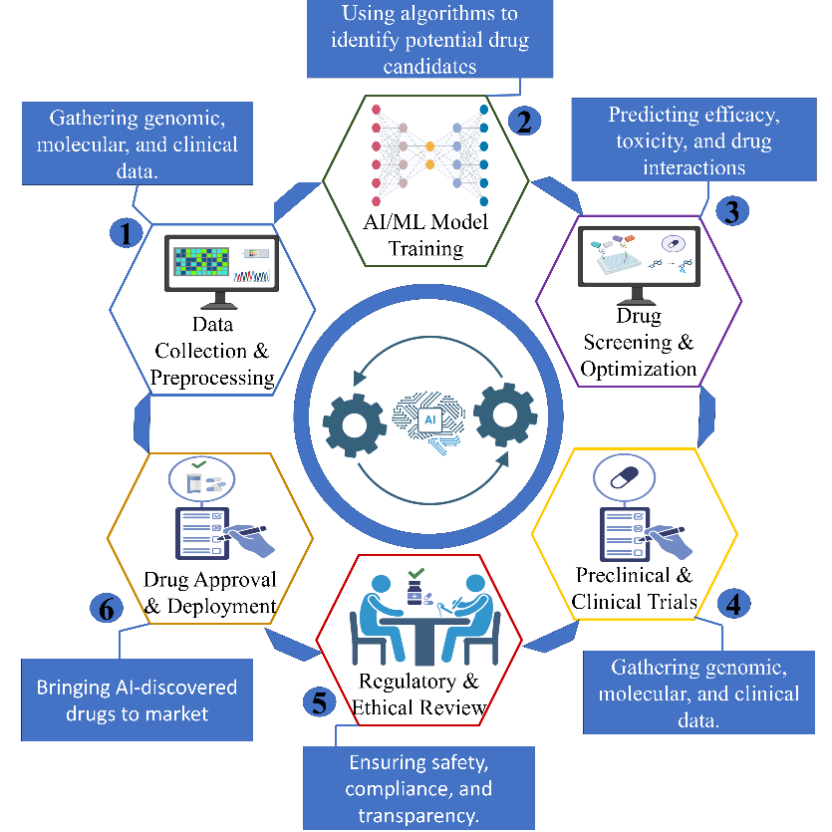
Graphical abstract: AI-powered neurological disease drug development
